# Geraniin Alleviates High‐Fat Diet‐Induced Atherosclerosis in *ApoE* ^−/−^ Mice

**DOI:** 10.1002/fsn3.70693

**Published:** 2025-07-25

**Authors:** Yaoyao Xie, Shihao Liu, Zhiheng Wei, Lisha Yu, Jianfeng Yu, Lu Xu, Honglin Jiang, Zhiliang Gu

**Affiliations:** ^1^ School of Biology and Food Engineering Changshu Institute of Technology Changshu China; ^2^ College of Pharmaceutical Science Soochow University Suzhou China; ^3^ School of Animal Sciences Virginia Polytechnic Institute and State University Blacksburg Virginia USA

**Keywords:** atherosclerosis, endothelial dysfunction, geraniin, network pharmacology, oxidative stress

## Abstract

Geraniin (GE), an ellagic tannin extracted from *Geranium wilfordii* , has been demonstrated to improve high‐fat diet‐induced metabolic disorders. This study aimed to investigate the effect of Geraniin on atherosclerosis and the underlying mechanism. In this study, we used the *ApoE* ^−/−^ mice as a model of atherosclerosis. We fed the *ApoE* ^−/−^ mice a high‐fat diet containing 1.25% cholesterol and treated them with or without 5 or 10 mg/kg body weight of GE for 10 weeks. Meanwhile, we combined network pharmacology analysis to explore how GE improves H_2_O_2_‐induced endothelial cell apoptosis and inflammatory response. Our results showed that GE treatment significantly reduced serum concentrations of lipids, oxidative stress damage, lipid deposition, and atherosclerotic plaque lesions in the *ApoE* ^−/−^ mice. Furthermore, a network pharmacology analysis indicated that the beneficial effect of GE treatment on atherosclerosis is closely related to its effect on oxidative stress, cell apoptosis, and inflammatory responses. We also showed that in H_2_O_2_‐treated HUVEC cells, GE promoted the release of NO, increased the activity of antioxidant enzymes, reduced endothelial cell apoptosis, reduced the production of inflammatory cytokines IL1β, IL6, and TNFα, and activated the Akt/eNOS/NO and GSK‐3β/Nrf2/HO‐1 signaling pathways. Taken together, these findings demonstrate that GE can alleviate high‐fat diet‐induced atherosclerosis in *ApoE* ^−/−^ mice by inhibiting oxidative stress‐induced endothelial cell apoptosis and inflammation.

## Graphical abstract

GE treatment significantly reduced serum concentrations of lipids, oxidative stress damage, lipid deposition, and atherosclerotic plaque lesions in the *ApoE* ^−/−^ mice. A network pharmacology analysis indicated that the beneficial effect of GE treatment on atherosclerosis is closely related to its effect on oxidative stress, cell apoptosis, and inflammatory responses. GE promoted the release of NO, increased the activity of antioxidant enzymes, reduced endothelial cell apoptosis, reduced the production of inflammatory cytokines IL1β, IL6, and TNFα, and activated the Akt/eNOS/NO and GSK‐3β/Nrf2/HO‐1 signaling pathways in H_2_O_2_‐treated HUVEC cells.

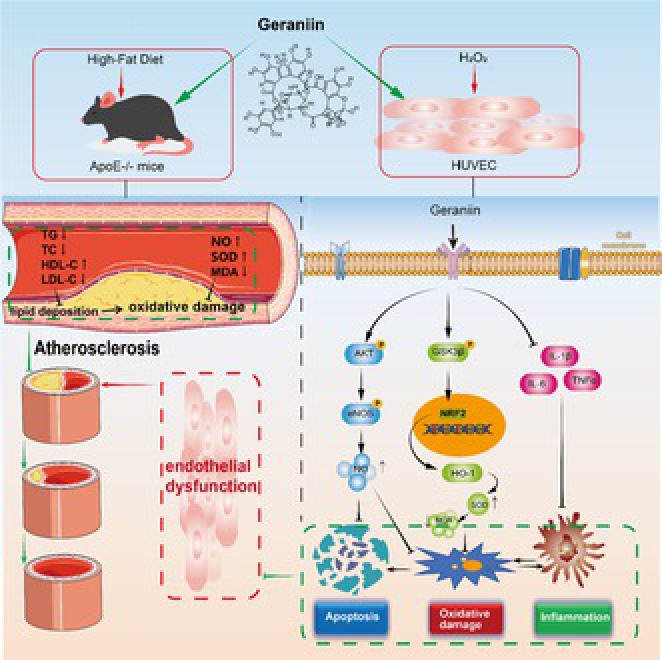

## 1 Introduction

In recent years, cardiovascular disease (CVD) has become a prominent health problem (Barbaresko et al. 2018; North and Sinclair 2012). According to the latest data from the World Health Organization (WHO), cardiovascular diseases (CVDs) remain the leading cause of death worldwide, accounting for an estimated 17.9 million deaths annually. In 2022, the global age‐standardized prevalence of CVD was approximately 10,000 cases per 100,000 population. This rate varied by region, with Central Asia reporting the highest prevalence at 11,342.6 per 100,000, and South Asia the lowest at 5881.0 per 100,000 (Mensah et al. 2023).

Atherosclerosis (AS) is a major CVD. Atherosclerosis occurs in the intima‐media of the arteries and is characterized by deposition of lipid plaques (Hopkins 2013). Rupture or erosion of atherosclerotic plaques leads to myocardial infarction and stroke, which account for 85% of all cardiovascular deaths (Poller et al. 2020). Atherosclerosis is one of the metabolic syndromes classified by the World Health Organization. If it occurs in the human body, the incidence of cardiovascular disease can be three times higher than the normal level (Herrington et al. 2016). Therefore, it is important to study the mechanisms and treatments of atherosclerosis.

Atherosclerosis can be attributed to obesity, hyperglycaemia, and hypertension, which contribute to oxidative stress (ROS) and chronic inflammation (Tietge 2014; Yuan et al. 2019). Endothelial cell dysfunction is considered to be the initiating factor of atherosclerosis (Balta 2021). Under pathological conditions, endothelial cell dysfunction causes the invasion of plasma lipids into the subcutaneous area, infiltration of monocytes and macrophages, and migration of vascular smooth muscle cells (Gimbrone Jr. and Garcia‐Cardena 2016; Tabas et al. 2015). Oxidative stress exerts negative effects on vascular cells, including endothelial dysfunction and activation, leukocyte migration and differentiation, vascular smooth muscle cell (VSMC) proliferation, and collagen degradation (Kattoor et al. 2017).

The most well‐established mechanism by which a high‐fat diet contributes to CVD is through its effect on lipid metabolism. Consuming large amounts of saturated fats, trans fats, and cholesterol leads to an increase in the levels of low‐density lipoprotein (LDL) cholesterol in the bloodstream (Heileson 2020). Elevated LDL cholesterol is a primary risk factor for the development of atherosclerosis, a condition characterized by the accumulation of fatty deposits (plaques) in the arterial walls. This accumulation of plaque narrows the arteries, reducing blood flow, and leading to an increased risk of heart attack, stroke, and peripheral artery disease (Sacks et al. 2017). Apolipoprotein E plays a crucial role in lipid metabolism by facilitating the clearance of cholesterol‐rich lipoproteins from the bloodstream. Apolipoprotein E (ApoE) knockout mice are a commonly used animal model in cardiovascular research. When the ApoE gene is knocked out, these mice develop impaired lipid metabolism, which leads to a significant increase in blood cholesterol levels, particularly low‐density lipoprotein cholesterol (LDL‐C), a known risk factor for atherosclerosis (Lo Sasso et al. 2016).

Geraniin (GE) is an ellagic tannin found in many traditional Chinese medicines such as *Geranium wilfordii* , *Phyllanthus urinaria* , and *Nephelium lappaceum* . GE has been shown to have antioxidant, anti‐inflammatory, anti‐hyperlipidemia, anti‐hypertension, and hepatoprotective actions (Aayadi et al. 2017; Cheng et al. 2020; Liu et al. 2016; Phang et al. 2019; Wang et al. 2015). Recent studies have demonstrated that GE can ameliorate diet‐induced metabolic risks by decreasing serum low‐density lipoprotein cholesterol (LDL‐c) and triglyceride (TG) levels, enhancing insulin sensitivity, and inhibiting lipid peroxidation (Cheng et al. 2020). Additionally, studies have shown that the administration of low doses of GE (3.125 and 6.25 mg/kg body weight) significantly reduced the production of advanced glycation end‐products (AGEs) in rats on a high‐fat diet and exhibited notable antihypertensive activity. This indicates that GE can ameliorate cardiovascular damage caused by metabolic disorders (Phang et al. 2019).

In this study, we investigated if GE has protective effects against high‐fat diet (HFD)‐induced atherosclerosis in *ApoE* ^−/−^ mice, which is a widely used mouse model of atherosclerosis (Zhang et al. 2021). We also studied the effect of GE on H_2_O_2_‐induced apoptosis and inflammation in HUVEC cells.

## 2 Materials and Methods

### 2.1 Materials

GE (98% purity) was obtained from Chroma Biotechnology Co. Ltd., Chengdu, China. Australian special fetal bovine serum (FBS) was acquired from Invitrogen Gibco, New York, USA. Dulbecco's Modified Eagle's Medium (DMEM) from Invitrogen Gibco, New York, USA. H_2_O_2_ was sourced from Union‐Bio Technology, Beijing, China. The MTT assay reagent was procured from Salarbio Molecular Technologies, Beijing, China.

Primary antibodies against eNOS (#AF0096), p‐eNOS (#AF3247), and Nrf2 (#AF0639) were acquired from Affinity Biosciences, Changzhou, China. Antibodies for TNF‐α (#WL01581), IL‐6 (#WL02841), and IL‐1β (#WLH3903) were sourced from Shenyang Wanlei Biological Technology Ltd., Shenyang, China. Antibodies for p‐Akt (SC‐514032), Akt (SC‐81434), p‐GSK‐3β (SC‐373800), GSK‐3β (SC‐81462), Bax (SC‐7480), Bcl‐2 (SC‐7382), HO‐1 (SC‐390991), Histone H3 (SC‐517576), and β‐Actin (SC‐47778) were purchased from Santa Cruz Biotechnology, CA, USA. LY294002 (an Akt inhibitor), SB216763 (a GSK‐3β inhibitor), and L‐NAME (an eNOS inhibitor) were procured from Beyotime Institute of Biotechnology, Shanghai, China.

### 2.2 Mice

All animal experiments adhered to the principles of experimental animal care established by Changshu Institute of Technology (Permit number: EAWEC202111). Six C57BL/6 male mice and eighteen *ApoE* ^−/−^ male mice (six weeks old) were acquired from Changzhou Cavens Laboratory Co. Ltd., Changzhou, China. Mice were housed in a Specific Pathogen Free (SPF) experimental environment, with the temperature set at 24°C ± 1°C, relative humidity controlled between 40% and 50%, and exposed to a 12‐h light–dark cycle. All mice were adaptively fed for one week. Then the C57BL/6 mice, as the control group, were fed a standard chow (MC10001, Moldiets Inc., China). The *ApoE* ^−/−^ mice were randomly assigned to one of three groups (*n* = 6 per group): (1) *ApoE* ^−/−^ model group; (2) GE 4 mg/kg body weight/day; (3) GE 8 mg/kg body weight/day. All *ApoE* ^−/−^ mice were fed a high‐fat diet (40 kcal% fat, D12108C, Research Diet Inc., USA) including 0.5% cholesterol for 10 weeks to establish an atherosclerosis model. GE was administrated intragastrically by oral gavage.

After undergoing a fasting period of 12 h, mice were sedated with ether, and blood samples were obtained from the retro‐orbital sinus. The collected blood was immediately placed on ice for 30 min and subsequently centrifuged at 3000 rpm for 10 min. The supernatant was then stored at −80°C. The serum levels of TC, TG, HDL‐C, LDL‐C, MDA, SOD, and NO in each mouse group were quantified using commercial assay kits, following the manufacturer's instructions (Nanjing Jiancheng Bioengineering Institute, Nanjing, China).

### 2.3 Pathological Detection of Atherosclerosis

Hematoxylin–eosin (HE), Oil Red O, and Masson's trichrome staining were performed to evaluate the degree of aortic disease. Thoracic aortas were dissected and fatty tissue on the outer layer of blood vessels was carefully removed. Then, the aortas were fixed in 4% paraformaldehyde and subsequently dehydrated with different concentrations of ethanol before being embedded in OCT compound. The entire aortas were stained with Oil Red O to facilitate plaque en face area analysis. Furthermore, tissue sections were stained with Hematoxylin and Eosin (H&E) and Masson's trichrome, and analyzed with ImageJ 1.46r software.

### 2.4 Network Pharmacology Analysis

The chemical structure and SMILES notation of Geraniin were obtained from the PubChem website (https://pubchem.ncbi.nlm.nih.gov/compound/3001497). Potential binding targets for Geraniin were acquired through SwissTargetPrediction (http://www.swisstargetprediction.ch/) (Gfeller et al. 2014), Comparative Toxicogenomics Database (http://ctdbase.org/) (Davis et al. 2021), PharmMapper Server (http://www.lilab‐ecust.cn/pharmmapper/index.html) (Wang et al. 2017), ChEMBL (https://www.ebi.ac.uk/chembl/) (Davies et al. 2015), and HERB (http://herb.ac.cn/) (Fang et al. 2021). Using the keyword “Atherosclerosis”, potential targets for atherosclerosis were obtained from DisGeNET (https://www.disgenet.org/) (Pinero et al. 2021), TTD (http://bidd.nus.edu.sg/BIDD‐Databases/TTD/TTD.asp) (Zhou et al. 2022), OMIM (https://www.omim.org/) (Amberger et al. 2015), DrugBank (https://www.drugbank.ca/) (Wishart et al. 2008), and GeneCard (https://www.genecards.org/) (Stelzer et al. 2016). The identified potential targets were input into the UniProt biodata platform (https://www.uniprot.org) for target annotation, with a species restriction to humans. After obtaining the genes corresponding to protein targets, duplicates were removed, and the resulting targets were saved.

The processed target genes were uploaded to the OE Cloud platform (https://cloud.oebiotech.com) to obtain intersecting target genes and create a Venn diagram. These intersecting target genes represent potential targets of GE for atherosclerosis treatment. The potential intersecting targets were uploaded into the STRING database (https://cn.string‐db.org/), with the organism parameter specified as *Homo sapiens* , for the creation of a protein–protein interaction (PPI) network. Subsequently, the node data of the PPI network were extracted and visualized using Cytoscape (version 3.7.0). The MCODE 2.0.3 plugin was utilized to isolate key target networks.

The intersecting target genes were submitted to the DAVID Bioinformatics platform (Sherman et al. 2022), with parameters set for the HOMO species, to obtain results of GO functional enrichment analysis including biological processes (BP), molecular functions (MF), cell components (CC), and KEGG pathway enrichment analysis. Visualize the results of GO functional enrichment and KEGG pathway analysis using the OE Cloud platform (https://cloud.oebiotech.com/task/).

### 2.5 Molecular Docking Prediction

The 3D structure of GE in SDF format was downloaded from the PubChem chemical information platform (https://pubchem.ncbi.nlm.nih.gov/compound/30‐01497). In AutoDockTools 1.5.7 (Morris et al. 2009), GE was selected as the small molecule ligand. The core target proteins, identified through network pharmacology, were selected as the protein receptors. The protein structures of core target proteins, in PDB format, were downloaded from the PDB database and preprocessed by removing water molecules and ligands, adding hydrogen atoms, and calculating charges. The AutoDockTools software was used to perform docking between the ligand and receptors. The affinity of compounds with the target was evaluated based on the obtained values of free binding energy, hydrogen bond count, and other factors. The optimal conformations of each receptor and ligand were visualized using PyMOL (Version 2.5, Schrödinger LLC).

### 2.6 Cell Culture and Treatments

Human umbilical vein endothelial cells (HUVECs), acquired from the Cell Bank of the Chinese Academy of Sciences, underwent cultivation in DMEM Medium enriched with 10% Fetal Bovine Serum (FBS) and a 1% mixture of Penicillin–Streptomycin (P/S). Cells were incubated in a constant temperature incubator at 37°C with a 5% CO_2_ atmosphere. For the purpose of experimentation, these cells were allocated into plates of various configurations: 6‐well, 12‐well, or 96‐well. To explore the protective effect of GE and its underlying mechanisms on HUVECs, the cells were allocated into the following groups: the control group, the H_2_O_2_ group (400 μM H_2_O_2_), and the protection group (4, 8, or 16 μM GE + 400 μM H_2_O_2_). To further confirm that geranin reduced H_2_O_2_‐induced HUVEC injury by activating the Akt/eNOS/NO and GSK‐3β/Nrf2/HO‐1 pathways, the cells were divided into the following groups: (1) control group, (2) H_2_O_2_ group (400 μM H_2_O_2_), (3) GE group (8 μM GE+400 μM H_2_O_2_), (4) LY294002 group (5 μM LY294002 + 8 μM GE+400 μM H_2_O_2_), (5) SB216763 group (10 μM SB216763 + 8 μM GE+400 μM H_2_O_2_), (6) L‐NAME group (100 μM L‐NAME+8 μM GE+400 μM H_2_O_2_).

### 2.7 Cell Viability Assay

To ascertain the viability of HUVECs, the MTT assay method was chosen. Initially, seeding the cells at a density of 10,000 cells per well into 96‐well plates was followed by incubation at 37°C for one day. Following this, the cells underwent treatment with GE ranging from 1 to 100 μM for a duration of 24 h, or were exposed to H_2_O_2_ alone (200–400 μM) for a period of 4 h to determine the most effective concentration. Additionally, a pre‐treatment with GE at concentrations of 4, 8, and 16 μM in serum‐free media was conducted for 24 h, before subjecting the cells to a 4‐h exposure to 400 μM H_2_O_2_. Then, 10 μL of MTT reagent was added to every well and allowed to incubate at 37°C for 4 h. Subsequently, the supernatant was removed, 100 μL of DMSO was introduced into each well, thorough mixing was ensured by shaking, and the absorbance was quantified at 490 nm with the aid of a microplate reader.

### 2.8 Flow Cytometry Analysis of Apoptosis

HUVECs were cultured in 6‐well plates. Following the aforementioned treatments, HUVECs were dissociated using trypsin without EDTA. Following detachment, cells were transferred to centrifuge tubes and subjected to three wash cycles with Phosphate‐Buffered Saline (PBS). The cells were then resuspended in 200 μL of Binding Buffer. For staining, 2 μL of Annexin V‐FITC and 2 μL of Propidium Iodide (PI) were introduced to the suspension. This mixture was then incubated at 37°C for a 20‐min period to allow for adequate staining. Flow cytometry analysis was conducted using a CytoFLEX (Beckman Coulter, USA) within an hour. These acquired data were analyzed using FlowJo version 10.4 software (TreeStar, based in Ashland, OR).

### 2.9 NO Assay

The effects of GE on NO release in HUVECs were studied. HUVECs were seeded in 24‐well plates and allowed to proliferate for a 24‐h period. After proliferation, cells were treated with varying concentrations of GE (4, 8, 16 μM) for an additional 24 h, followed by a 4‐h treatment with 400 μM H_2_O_2_. To measure nitrite levels, a 160 μL sample of the cell culture medium was collected from each well. Nitrite concentration, a proxy for NO production, was quantified following the assay kit manufacturer's guidelines. Quantification involved measuring absorbance at 550 nm, using a sodium nitrite standard curve for calibration.

### 2.10 Determination of MDA and SOD Activities

Activities of MDA and SOD in HUVECs were measured to assess oxidative stress in cells using commercial kits. Briefly, HUVECs were harvested and centrifuged at 1000 × *g* for 3 min. The cells were resuspended with 500 μL PBS and then lysed by sonication. Following centrifugation, the supernatant was collected for MDA and SOD detection. Prepared samples were mixed with the working solution and incubated at 37°C for 15 min. Absorbance at 532 nm (for MDA) and 520 nm (for SOD) was measured with a microplate reader.

### 2.11 Western Blot Analysis

Protein extraction from HUVECs was achieved using RIPA lysis buffer, enhanced with 0.1 mM Phenylmethylsulfonyl fluoride (PMSF) and a cocktail of phosphatase inhibitors to ensure the stability and integrity of the proteins. The concentration of extracted proteins was quantified employing the BCA Protein Assay Kit (Beyotime Biotechnology, Shanghai, China). For the separation of these proteins, SDS‐Polyacrylamide Gel Electrophoresis (SDS‐PAGE) was utilized, followed by their transfer to Polyvinylidene Difluoride (PVDF) membranes (Roche Diagnostics Limited, Shanghai, China). To block non‐specific attachment, membranes were treated with 5% skim milk as a blocking agent for a duration of 2 h at room temperature. Following this, an overnight exposure to primary antibodies, designed to target p‐Akt, Akt, p‐GSK‐3β, GSK‐3β, p‐eNOS, eNOS, Nrf2, HO‐1, Bax, Bcl‐2, and β‐actin, was performed at 4°C. Following the primary antibody incubation, membranes were thoroughly washed three times with TBST (Tris‐buffered saline with Tween20) and subsequently incubated with HRP‐conjugated secondary antibodies targeted against rabbit or mouse Immunoglobulin G (IgG) at a dilution of 1:10000 for 2 h at room temperature. Detection of the protein bands was facilitated using an electrochemiluminescence (ECL) detection kit, and quantitative analysis of the protein expression levels was performed using Image J software for densitometric analysis.

### 2.12 Statistical Analysis

The experimental outcomes are expressed as the mean and the standard deviation (Mean ± SD). Multiple comparisons were analyzed through one‐way ANOVA, followed by a subsequent Tukey's post hoc test. The statistical analysis was carried out with SPSS software (version 19.0). *p*‐value < 0.05 was interpreted as indicating a significant difference.

## 3 Results

### 3.1 Effects of Geraniin on Plasma Lipid Levels in HFD‐Fed *ApoE* ^−/−^mice

**FIGURE 1:**
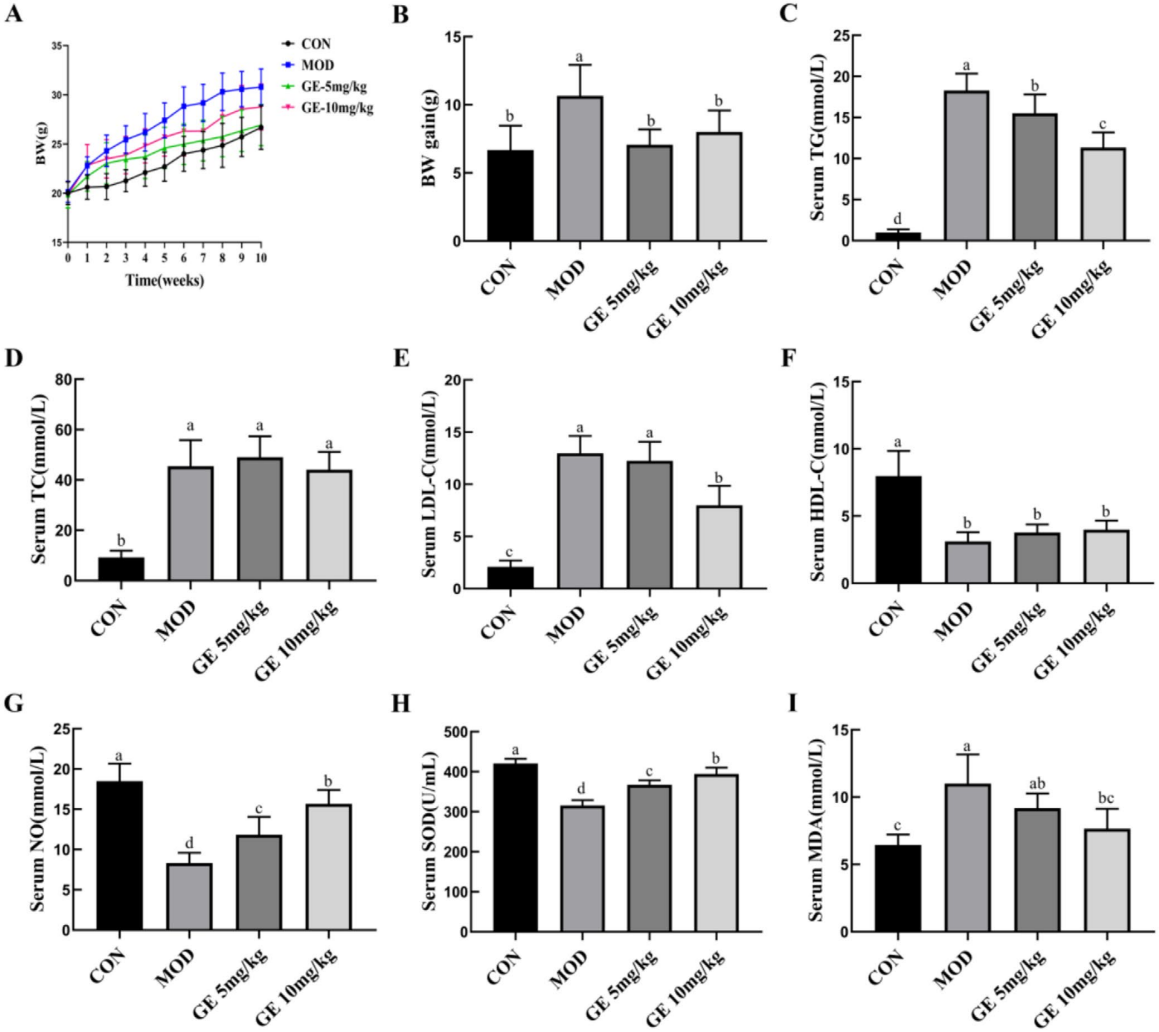
Effects of Geraniin on body weight, serum lipids, and oxidative stress index in HFD‐fed *ApoE* ^−/−^ mice. *ApoE* ^−/−^ mice were treated with GE or CMC‐Na for 10 weeks during high‐fat diet feeding. Serum was collected to assess lipid metabolism and oxidative stress levels. (A) Body weight curves. (B) The average weight gain. (C) Serum TG level. (D) Serum TC level. (E) Serum LDL‐C level. (F) Serum HDL‐C level. (G) Serum NO level. (H) Serum MDA level. (I) Serum SOD level. Data are presented as means ± SD (*n* = 6). Various letters indicate significant differences (*p* < 0.05) among groups.

After a 10‐week high‐fat diet regimen, *ApoE* ^−/−^ mice demonstrated a significant increase in body weight compared to the control group. However, the body weight of *ApoE* ^−/−^ mice treated with GE (5 mg/kg or 10 mg/kg) was significantly reduced compared with that of the *ApoE* ^−/−^ model group (Figure 1A,B). Serum levels of TG, TC, and LDL‐C were remarkably higher, and serum HDL‐C levels were significantly lower in the *ApoE* ^−/−^ model group than in the control group, (Figure 1C–F). GE treatment decreased serum TG (Figure 1C) and LDL‐C levels (Figure 1E) in *ApoE* ^−/−^ mice. However, GE had no effect on serum TC level (Figure 1D) and HDL‐C level (Figure 1F). Compared with the control group, serum NO levels in the *ApoE* ^−/−^ model group were significantly lower. However, GE treatment increased serum NO production (Figure 1G). As shown in Figure 1H,I, high fat‐diet fed *ApoE* ^−/−^ mice displayed notable oxidative damage, and GE treatment significantly decreased serum MDA but increased SOD activity.

### 3.2 Geraniin Alleviated the Atherosclerotic Lesions in the Aortas of *ApoE* ^−/−^ Mice

**FIGURE 2:**
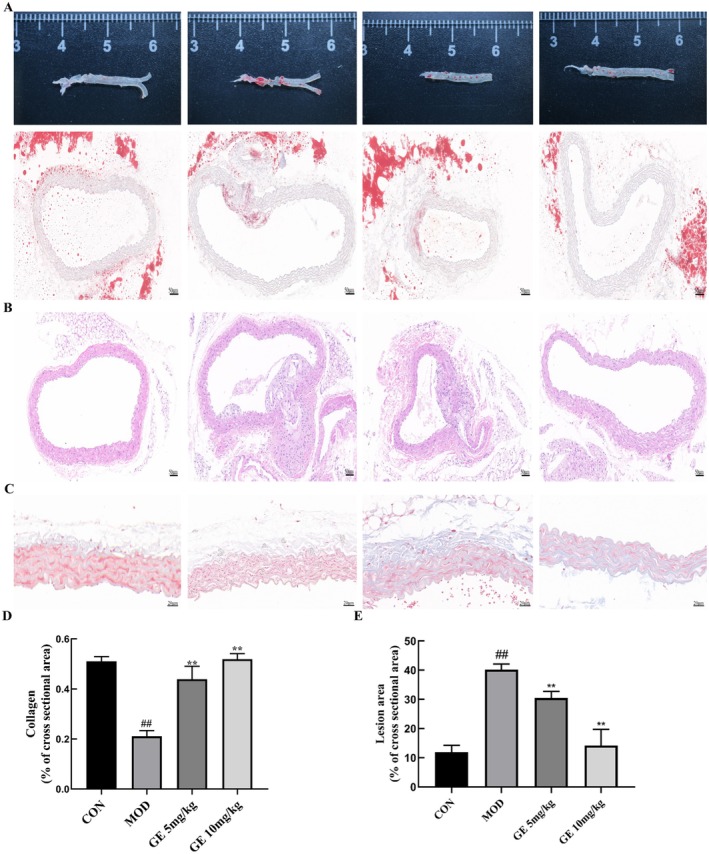
Effects of Geraniin on the formation of atherosclerotic lesion in HFD‐fed *ApoE* ^−/−^ mice. *ApoE* ^−/−^ mice were treated with GE or CMC‐Na for 10 weeks during high‐fat diet feeding. Thoracic aortas were collected for endothelial function assessment. (A) Oil red O staining of the atherosclerotic plaques in aortic roots. (B) En face oil‐red O staining of the atherosclerotic plaques in aortas. (C) Representative images of aortic sections stained with H&E. (D) Masson staining of aortic tissue. (E) Quantitation of the atherosclerotic plaques in aortas. (F) Quantification of the Masson staining area. Data are presented as means ± SD (*n* = 6). Various letters indicate significant differences (*p* < 0.05) among groups.

To evaluate the effects of GE on atherosclerotic formation, we investigated the aortic lesion burden. Oil Red O staining indicated a significant decrease in lipid accumulation in GE‐treated *ApoE* ^−/−^ mice relative to the *ApoE* ^−/−^ model group (Figure 2A,B). Furthermore, the area of arterial plaque in atherosclerotic mice receiving GE treatment was notably smaller than in those without treatment (Figure 2C). Quantitative evaluation revealed that 10 mg/kg GE reduced the size of the lesion area by 62.4% compared with the untreated *ApoE* ^−/−^ model group (Figure 2E). Masson trichrome staining revealed reduced collagen content in HFD‐fed *ApoE* ^−/−^ mice while increased collagen formation in HFD‐fed *ApoE* ^−/−^ mice administered with GE compared with control mice (Figure 2D,F), suggesting that GE can increase collagen content in plaque and thereby enhance plaque stability.

### 3.3 Network Pharmacology Analysis

**FIGURE 3:**
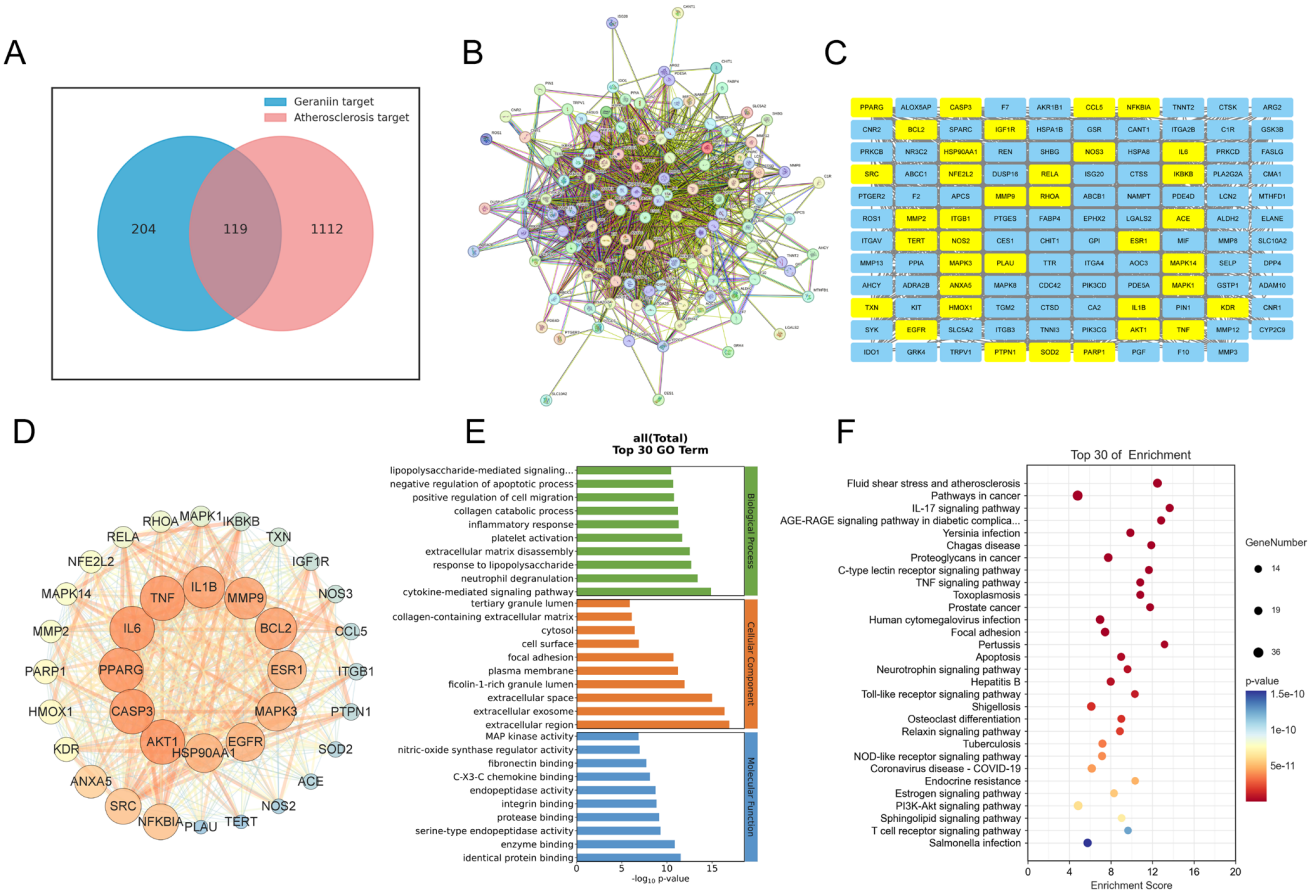
Identification of atherosclerosis‐related genes potentially targeted by Geraniin through a network pharmacology analysis. (A) Venn diagram of Geraniin‐targeted genes and atherosclerosis‐related genes. (B) Protein–protein interaction (PPI) network of potential GE‐targeted atherosclerosis‐related genes. (C) Selecting key targets from potential GE‐targeted atherosclerosis‐related genes. (D) Network of key Geraniin targets in treating atherosclerosis. (E) GO functional enrichment analysis of potential GE targets. (F) KEGG pathway enrichment analysis of potential GE targets.

We predicted GE's potential target genes through SwissTargetPrediction, Comparative Toxicogenomics Database (CTD), PharmMapper, ChEMBL, and HERB databases. After unification, merging, and removing duplicates through UniProt, 323 target genes were predicted for GE. We collected genes associated with atherosclerosis from DisGeNET, TTD, OMIM, DrugBank, and GeneCard databases. After unification, merging, and removing duplicates through UniProt, 1231 atherosclerosis‐related genes were obtained. We uploaded the two sets of genes to the OE Cloud platform to create a Venn diagram. Shown in Figure 3A, 119 atherosclerosis‐related genes could be targeted by GE.

The 119 potential target genes were imported into the STRING protein–protein interaction database to construct a visual PPI network (119 nodes, 1478 edges), resulting in a network diagram illustrating the interactions among various protein targets (Figure 3B). To further filter out key target genes, we exported node data from the PPI network and visualized it using Cytoscape 3.9.0 software. In Cytoscape 3.9.0, we used the MCODE 2.0.3 plugin with default parameters to identify a key target network composed of 36 nodes and 497 edges (Figure 3C,D). These key targets are closely associated with inflammatory responses (such as TNF, IL‐6, IL‐1β, NFKBIA), oxidative stress (such as AKT1, NFE2L2, HOMX1, MAPK), and cellular apoptosis (CASP3, BCL2, MMP9).

In addition, we conducted GO function and KEGG pathway enrichment analyses on the 119 potential target genes of GE. As shown in Figure 3E, the BP terms were primarily associated with cytokine‐mediated signaling pathway, neutrophil degranulation, and response to lipopolysaccharide. The main CC terms included the extracellular region, extracellular exosome, and extracellular space. The predominant Molecular Function (MF) terms included identical protein binding, enzyme binding, and serine‐type endopeptidase activity. KEGG pathway analysis revealed significant enrichment of these potential target proteins in pathways, including fluid shear stress and atherosclerosis, AGE‐RAGE signaling pathway in diabetic complications, IL‐17 signaling pathway, apoptosis, TNF signaling pathway, Toll‐like receptor signaling pathway, and PI3K‐Akt signaling pathway (Figure 3F).

### 3.4 Molecular Docking Prediction and Visualization

**FIGURE 4:**
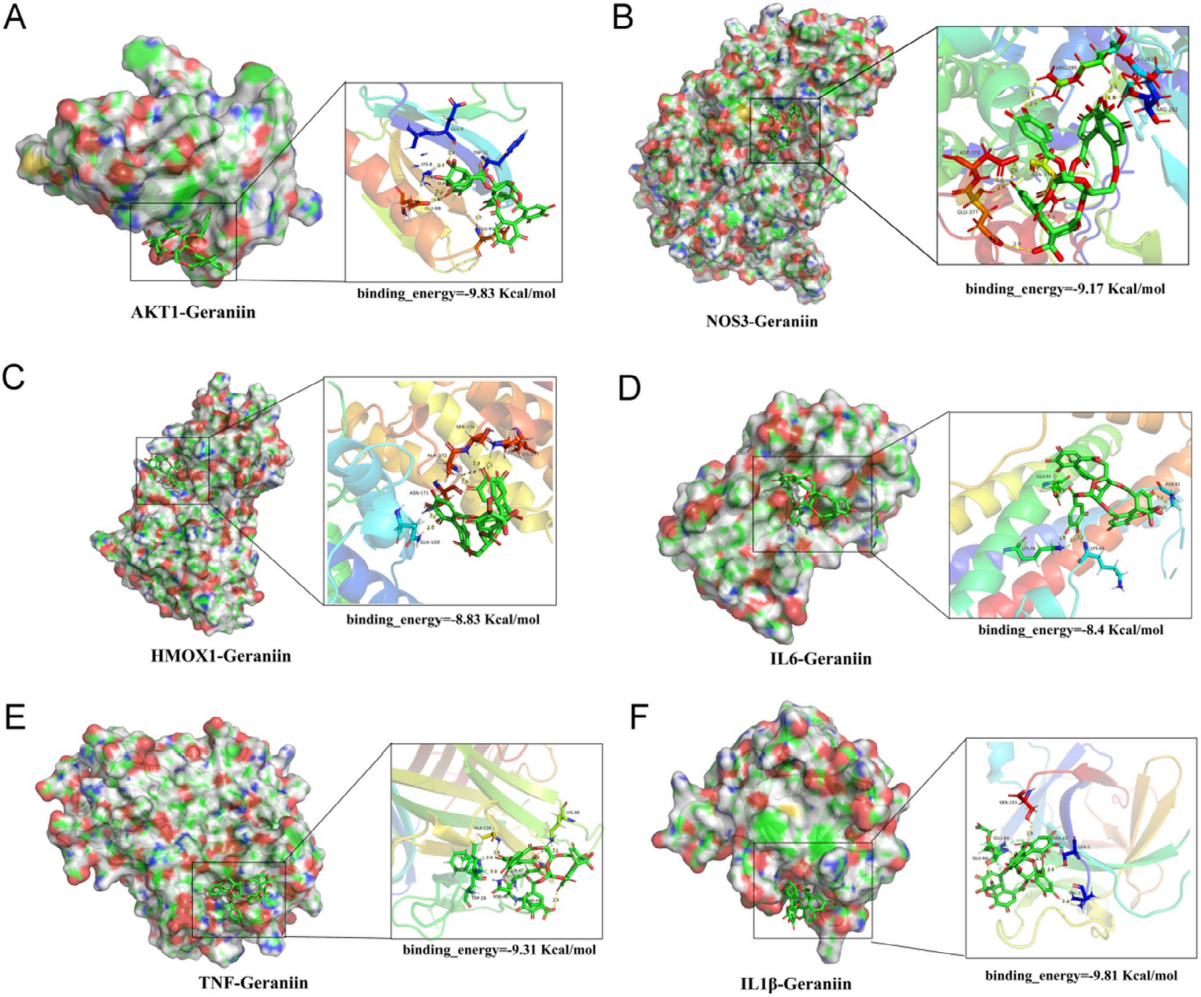
Docking and visualization of Geraniin with protein targets. (A) Optimal molecular docking conformation of Geraniin with AKT1 protein. (B) Optimal molecular docking conformation of Geraniin with NOS3 protein. (C) Optimal molecular docking conformation of Geraniin with HMOX1 protein. (D) Optimal molecular docking conformation of Geraniin with IL6 protein. (E) Optimal molecular docking conformation of Geraniin with TNF protein. (F) Optimal molecular docking conformation of Geraniin with IL1β protein.

Based on the functions of key GE target genes and the GO and KEGG enrichment analysis of these genes, the mechanisms by which GE alleviates atherosclerosis may involve pathways related to inflammation, oxidative stress, and apoptosis. Therefore, we selected genes (IL1β, TNF, IL6, AKT1, NOS3, HMOX1) closely associated with these pathways from the key GE targets for molecular docking verification. The binding ability between molecules and ligands is evaluated by the magnitude of free binding energy, with smaller free binding energy indicating tighter binding. Generally, a molecular docking binding energy less than −1 kcal/mol (1 cal = 4.4 J) indicates binding activity, and a binding energy less than −5 kcal/mol indicates good binding activity. We used Autodock 4.2 to perform docking predictions between GE and each protein, selecting the optimal binding conformations for visualization (Figure 4A–F). The binding energies between GE and AKT1, NOS3, HMOX1, IL1β, IL6, and TNF were −9.83, −9.17, −8.83, −8.4, −9.31, and −9.81 kcal/mol, respectively, indicating excellent binding activity between GE and these protein molecules.

### 3.5 Geraniin Protected HUVECs Against H_2_O_2_ ‐Induced Injury

**FIGURE 5:**
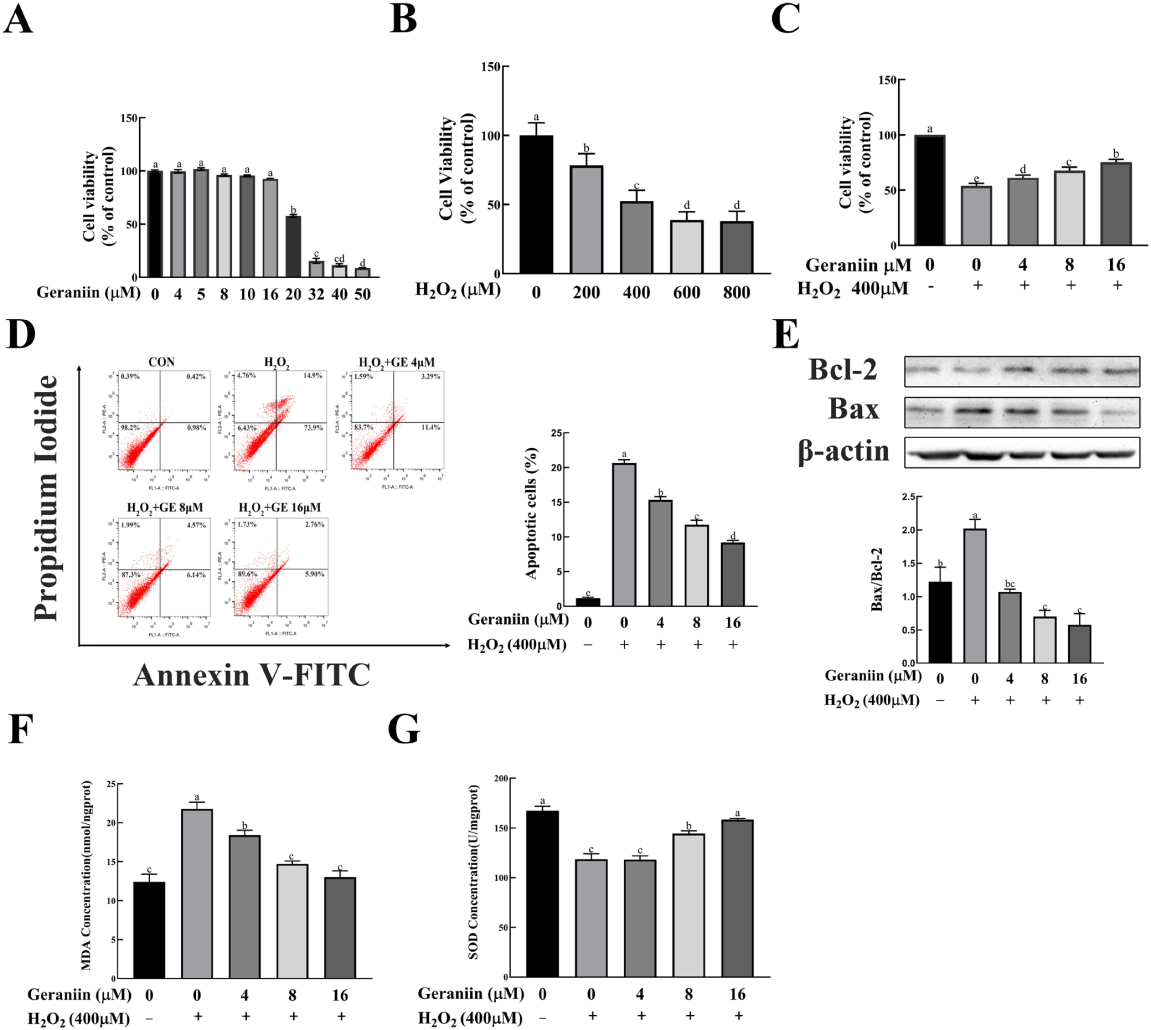
Effects of Geraniin on H_2_O_2_‐induced cytotoxicity, apoptosis, and oxidative stress in HUVEC cells. HUVEC cells were incubated with the indicated concentrations of Geraniin or H_2_O_2_ or both for 24 h or 4 h before viability, apoptosis, Bcl‐2 and Bax expression, and MDA and SOD contents were analyzed. (A) Effect of Geraniin on viability of HUVEC cells. (B) Effect of H_2_O_2_ on viability of HUVEC cells. (C) Effect of Geraniin on H_2_O_2_‐triggered cell death. (D) Flow cytometry of H_2_O_2_‐induced apoptosis. (E) Effects of Geraniin on the expression of Bax and Bcl‐2 in H_2_O_2_‐induced HUVEC cells. (F, G) Effects of Geraniin on the MDA and SOD activity in H_2_O_2_‐induced HUVEC cells. Data are presented as mean ± SD (*n* = 3). Distinct letters denote significant differences (*p* < 0.05) among groups.

The aforementioned animal experiments and network pharmacology analysis indicate that the mechanism of action of GE on atherosclerosis may be closely related to its effect on oxidative stress, apoptosis, and inflammatory responses. We validated these effects of GE in an H_2_O_2_‐induced HUVEC cell damage model. To assess the cytotoxicity of H_2_O_2_ and GE on HUVECs, we treated cells with different concentrations of H_2_O_2_ (100⁓1000 μM) for 4 h and different concentrations of GE (1⁓200 μM) for 24 h. As depicted in Figure 5A, treating HUVECs with GE at concentrations of 4, 8, or 16 μM for 24 h did not markedly impact cell viability. Meanwhile, we found that the cell viability of HUVECs was concentration‐dependently decreased when treated with 200–1000 μM H_2_O_2_ for 4 h. These data indicated that 400 μM was an appropriate H_2_O_2_ concentration for intervention (Figure 5B). Pretreatment of HUVECs with 4 to 16 μM GE effectively protected against 400 μM H_2_O_2_‐induced cell injury in a dose‐dependent manner (Figure 5C). Excessive apoptosis of HUVEC cells may destroy the structural integrity of endothelial cells and increase plaque vulnerability in the process of atherosclerosis. Thus, flow cytometry was performed to test the effect of GE on the apoptosis of endothelial cells in vitro. As shown in Figure 5D, GE significantly decreased apoptosis in HUVECs induced by H_2_O_2_ treatment. Consistent with the Annexin V‐FITC/PI staining assays, Western blotting results showed that the ratio of Bax/Bcl‐2 in HUVEC cells was significantly increased by H_2_O_2_ treatment, while it was decreased in a concentration‐dependent manner by GE intervention (Figure 5E).

### 3.6 Geraniin Reduced H_2_O_2_ ‐Induced Oxidative Stress in HUVECs

To evaluate the effect of GE on H_2_O_2_‐induced antioxidant capacity of HUVEC cells, we measured MDA content and SOD activity in the cells. As revealed in Figure 5F,G, H_2_O_2_ resulted in oxidative stress in HUVECs, accompanied by increased MDA content and decreased SOD activity. However, GE reversed these H_2_O_2_‐induced changes, exerting a strong antioxidant effect.

### 3.7 Geraniin Activated the Akt/ eNOS /NO Signaling Pathway

**FIGURE 6:**
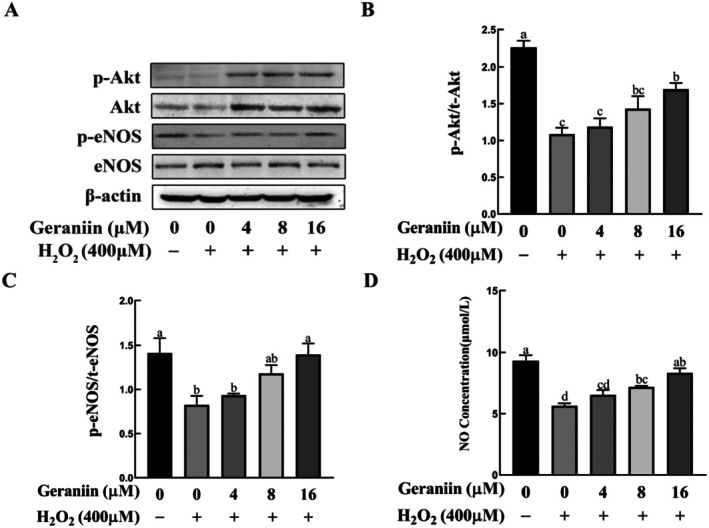
Effects of geraniin on the phosphorylation of Akt and eNOS, and NO geraniin generation in H_2_O_2_‐induced HUVEC cells. (A) Representative western blots showing protein expressions of p‐Akt and p‐eNOS. (B) Ratio of p‐Akt/t‐Akt. (C) Ratio of p‐eNOS/t‐eNOS. (D) NO concentration in HUVEC medium. Data are presented as mean ± SD (*n* = 3). Distinct letters denote significant differences (*p* < 0.05) among groups.

Activation of the Akt/eNOS/NO pathway plays a vital role in protecting endothelial cells and inhibiting cell apoptosis (Dong et al. 2021). Network pharmacology and molecular docking analyses indicated that AKT1 and NOS3 (also known as eNOS) play crucial roles in GE alleviating atherosclerosis. We therefore determined whether GE activates this signaling pathway in HUVEC cells. Western blotting was performed to evaluate the p‐Akt, Akt, p‐eNOS, and eNOS expression levels in HUVECs. The levels of p‐Akt/Akt and p‐eNOS/eNOS ratios significantly declined in cells exposed to H_2_O_2_, in contrast to the untreated counterparts. Pretreatment with 16 μM GE increased the ratios of p‐Akt/Akt and p‐eNOS/eNOS in H_2_O_2_‐treated HUVEC cells (Figure 6A–C). Meanwhile, medium NO concentrations were significantly lower in H_2_O_2_‐treated than in untreated HUVEC cells, and this difference was reversed by 8 and 16 μM GE (Figure 6D). These results indicate that GE may mitigate H_2_O_2_‐induced apoptosis in HUVEC cells by activating the Akt/eNOS/NO pathway.

### 3.8 Geraniin Activated the GSK‐3β/ Nrf2 /HO‐1 Signaling Pathway

**FIGURE 7:**
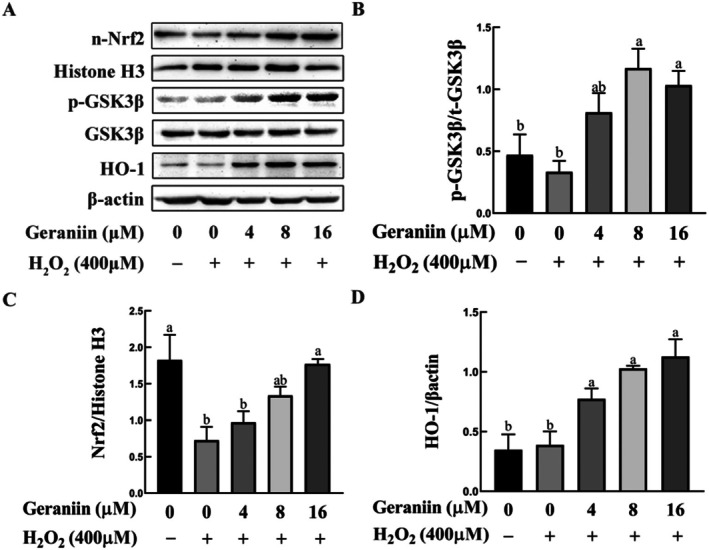
Effects of geraniin on the phosphorylation of GSK‐3β and the expression of Nrf2 and HO‐1 in H_2_O_2_‐induced HUVEC cells. (A) Representative western blots showing protein expressions of p‐GSK3β, n‐Nrf2, and HO‐1. (B) Ratio of p‐GSK3β/t‐GSK3β. (C) Ratio of n‐Nrf2/Histone H3. (D) Ratio of HO‐1/β‐Actin. Data are presented as mean ± SD (*n* = 3). Distinct letters denote significant differences (*p* < 0.05) among groups. The consistent β‐Actin bands in Figures 5E and 7A derive from parallel detection of the same sample batch. All data were independently validated through biological replicates.

The results of network pharmacology analysis also suggest that the two antioxidant‐related genes, NFEL2 and HMOX1, may play vital roles in the process of GE improving atherosclerosis. Our experiments have validated this point, as shown by Western blotting analyses (Figure 7C,D), the expression of nuclear Nrf2 in HUVEC cells was decreased 2.5‐fold by H_2_O_2_ in comparison to that in untreated cells while GE upregulated nuclear Nrf2 and HO‐1 in a concentration‐dependent manner. Given that GSK‐3β is a recognized negative regulator of Nrf2, we explored the possibility of GE inducing phosphorylation of GSK‐3β at Ser9 (Cuadrado 2015). It was observed that exposure to 8 μM of GE induced a roughly 1.7‐fold increase in the phosphorylation levels of GSK‐3β (Ser9) in HUVEC cells subjected to H_2_O_2_ stress (Figure 7B).

### 3.9 Geraniin Ameliorated H_2_O_2_ ‐Induced Apoptosis in HUVEC Cells via the Akt/eNOS/NO and GSK‐3β/Nrf2/HO‐1 Pathways

**FIGURE 8:**
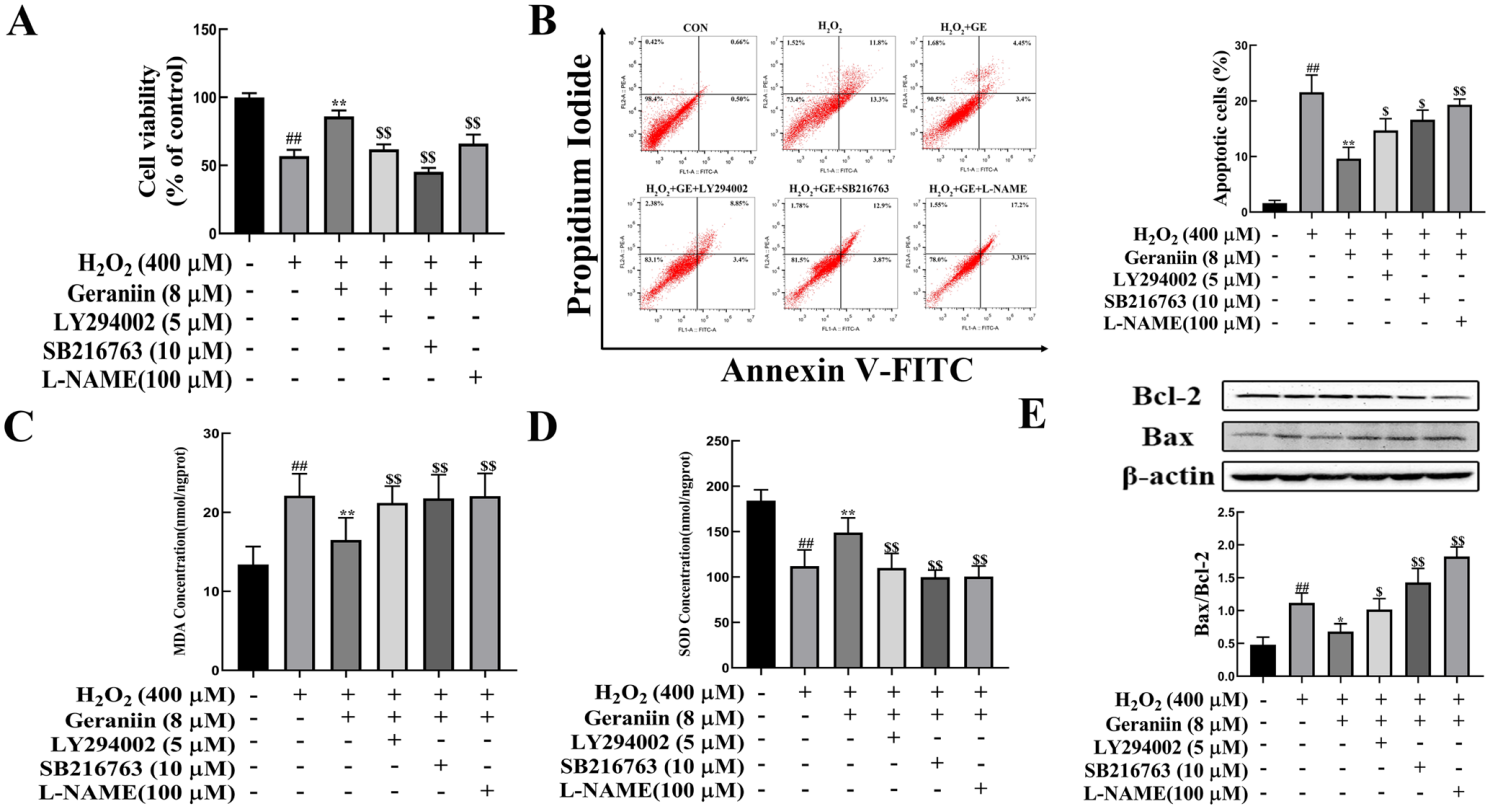
Geraniin activation of the Akt/eNOS/NO and GSK‐3β/Nrf2/HO‐1 signaling in HUVEC cells. HUVECs were treated with Geraniin (8 μM), LY294002 (5 μM), SB216763 (10 μM), and L‐NAME (100 μM) for 24 h and stimulated with H_2_O_2_ for 4 h. (A) Cell viability. (B) V‐FITC/PI staining and quantitative analysis of apoptotic cells. (C) Medium MDA concentration. (D) Medium SOD concentration. (E) Representative western blots showing protein expressions of Bax and Bcl‐2 and quantification of Bax and Bcl‐2. Data are presented as mean ± SD (*n* = 3). Distinct letters denote significant differences (*p* < 0.05) among groups.

To confirm that GE can exert a protective effect on vascular endothelial cells by activating the Akt/eNOS/NO and GSK‐3β/Nrf2/HO‐1 pathways, we used the Akt inhibitor (LY294002), GSK‐3β inhibitor (SB216763) and eNOS inhibitor (L‐NAME) to block these pathways. Figure 8A–C show that pretreatment with GE significantly enhanced cell survival and reduced apoptosis. However, the beneficial effects of GE were markedly reduced following the administration of LY294002, SB216763, and L‐NAME to the cells. Meanwhile, GE‐induced increases in SOD activity and decreases in MDA were also blocked by LY294002, SB216763, and L‐NAME treatment (Figure 8C,D). The ratio of Bax/Bcl‐2 significantly decreased after treatment with these inhibitors (Figure 8E), suggesting the protective effects of GE on HUVECs were inhibited. Collectively, these results indicated that the Akt/eNOS/NO and GSK‐3β/Nrf2/HO‐1 signaling pathways mediate the protective effects of GE against H_2_O_2_‐induced oxidative damage in HUVECs.

### 3.10 Geraniin Reduces the Expression of Inflammatory Cytokines in H_2_O_2_ ‐Induced HUVECs

**FIGURE 9:**
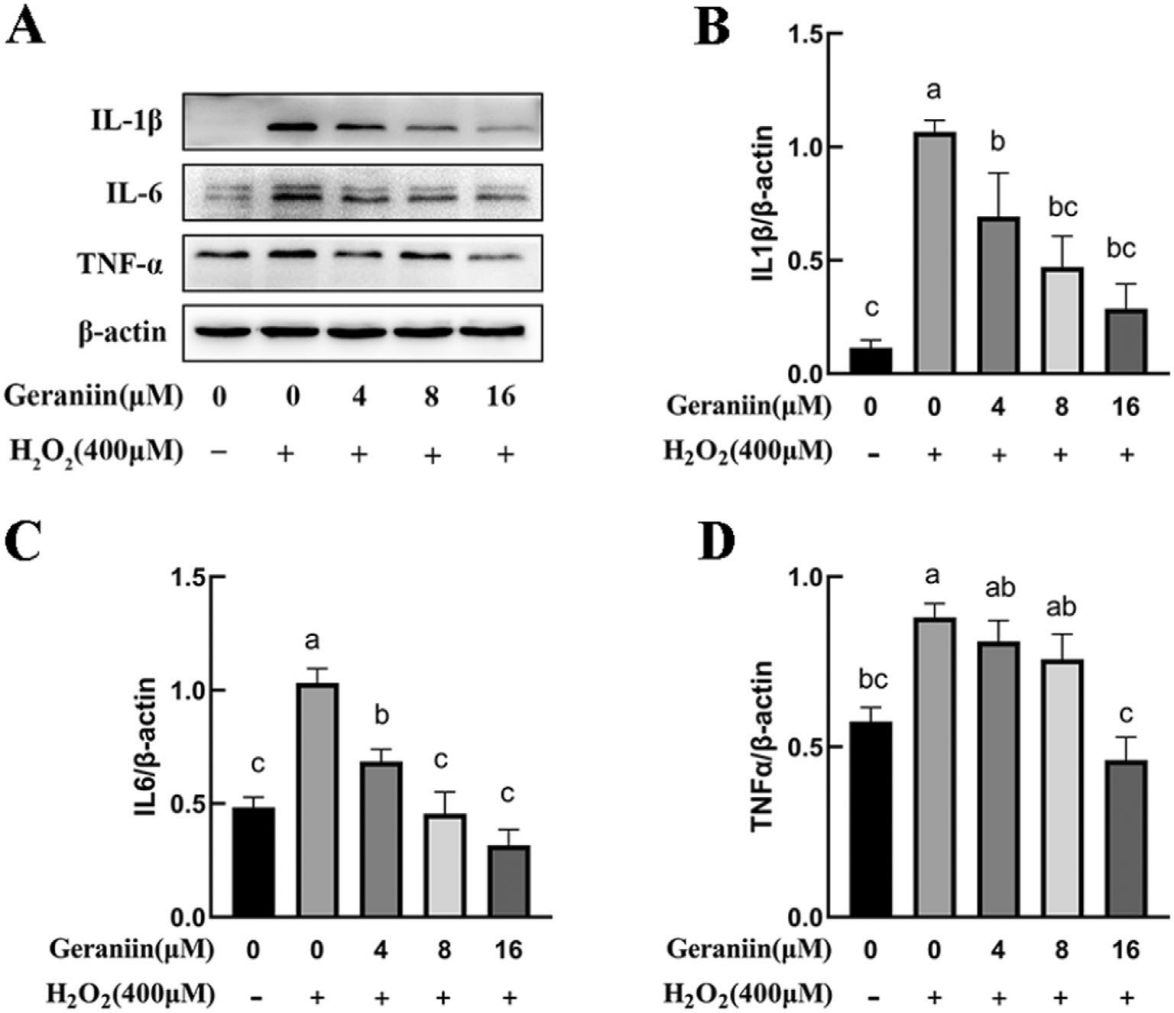
Effects of Geraniin on the expression of IL1β, IL6, and TNFα proteins in H_2_O_2_‐induced HUVEC cells. (A) Representative western blots showing protein expressions of IL1β, IL6, and TNFα. (B) Ratio of IL1β/β‐Actin. (C) Ratio of IL6/β‐Actin. (D) Ratio of TNFα/β‐Actin. Data are presented as mean ± SD (*n* = 3). Distinct letters denote significant differences (*p* < 0.05) among groups.

Inflammatory response is a critical factor driving the progression of atherosclerotic lesions. Studies have shown that inflammatory cytokines and other acute response molecules released by endothelial cells during inflammation exacerbate endothelial dysfunction (North and Sinclair 2012). In conjunction with the earlier network pharmacology analysis results, we conducted Western blot analysis of the three inflammatory factors, IL1β, IL6, and TNFα, in HUVEC cells (Figure 9A). The results showed that compared to the untreated group, H_2_O_2_ treatment significantly increased the expression of IL1β, IL6, and TNFα in HUVEC, exacerbating the intracellular inflammatory response. After treating the H_2_O_2_‐exposed group with different concentrations of GE, the expression of IL1β, IL6, and TNFα in the cells exhibited a concentration‐dependent significant decrease (Figure 9B–D), indicating that GE can improve the inflammation induced by H_2_O_2_ in HUVEC cells.

## 4 Discussion

Dyslipidemia is a major risk factor for vascular diseases associated with atherosclerosis (Helkin et al. 2016). Lowering blood lipid levels has always been an important intervention to alleviate the progression of atherosclerosis (Mourikis et al. 2020). A study in HFD‐fed rats showed that GE exhibited protective effects against dyslipidemia by decreasing the levels of TG, non‐HDL, and total cholesterol. In alignment with prior research, our study demonstrated that treating HFD‐fed *ApoE* ^−/−^ mice with GE significantly improved serum lipid profiles, including reduced levels of TC, TG, and LDL‐C, and an elevated level of HDL‐C. Moreover, our data demonstrated that GE inhibited aortic lipid deposition and slowed the progression of atherosclerosis.

The choice of 5 mg/kg and 10 mg/kg as dosages for geraniin in our study is based on the principles of dose‐dependent response. These dosages are within a range commonly used in preclinical studies for testing the therapeutic effects of natural compounds, especially polyphenols. Historical studies on geraniin and similar natural products, such as curcumin and resveratrol, demonstrate that these doses are likely to provide measurable effects on inflammation, oxidative stress, and lipid metabolism without significant toxicity (Lv et al. 2020). The 5 mg/kg dose may target initial therapeutic effects, while the 10 mg/kg dose could potentially enhance these effects, making this approach an appropriate balance for assessing geraniin's efficacy in treating high‐fat diet‐induced atherosclerosis.

Lipid metabolism disorders can lead to increased production of reactive oxygen species (Li and Yang 2018). In endothelial cells, excessive ROS mediates the production and secretion of cytokines, which promote monocytes to adhere to the intima and differentiate into macrophages (Rendra et al. 2019). Macrophages engulf lipoprotein and then transform into foam cells at the lesion site, promoting the early formation of atherosclerosis (Incalza et al. 2018; Thomas et al. 2008). A more recent study showed that tannin compounds have antioxidant and anti‐inflammatory activities (Petroski and Minich 2020). Thus, we speculated that the protective effect of geraniin on arterial blood wall may involve its antioxidant and anti‐inflammatory properties. GE exerts its therapeutic effects on liver injury and kidney injury via scavenging free radicals, inhibiting lipid peroxidation, and improving the activities of antioxidant enzymes (Jiang et al. 2016). Levels of MDA and SOD reflect the oxidative damage and antioxidant damage, respectively (Del Rio et al. 2005; den Hartog et al. 2003). In this study, a significant increase in the levels of MDA, the final product of oxidative stress, was observed in *ApoE* ^−/−^ mice fed a high‐fat diet. Also, the antioxidant enzyme SOD was substantially increased, indicating that atherosclerosis is associated with oxidative stress. GE administration significantly reduced ROS production by enhancing SOD activity and decreasing MDA generation. Nitric oxide (NO), a crucial endothelium‐derived relaxation factor, is essential for maintaining vascular tone and reactivity (Vidanapathirana et al. 2021). NO activity is an important indicator of endothelial dysfunction. This study showed that the serum NO content in *ApoE* ^−/−^ mice was significantly lower than that in normal mice, and that GE treatment significantly increased the level of NO. Therefore, the effects of GE on vascular endothelial contraction and relaxation may be an important mechanism of its protective effect against vascular endothelial dysfunction. Collectively, these findings indicate that GE protects against endothelial injury, thereby ameliorating oxidative stress and atherosclerosis.

To further explore the mechanism by which GE improves atherosclerosis, we conducted preliminary investigations using network pharmacology and molecular docking techniques. Network pharmacology is a comprehensive bioinformatics analysis technique that integrates pharmacology, systems biology, network informatics, and computer science (Pereira and Aires‐de‐Sousa 2018). By constructing protein–protein interaction networks, gene expression profiles, and biological pathway data, network pharmacology can reveal complex interactions between drugs and cells and biomolecules. This not only aids in a deeper understanding of the drug's mechanism of action but also provides more targeted approaches for drug design and development. Molecular docking is a technique that predicts the interaction between biological macromolecules and small molecule ligands by assessing their matching degree through computer models (Chen et al. 2020). This technology can reduce the randomness in drug research, increase the success rate of drug screening, and make drug screening cost‐effective and widely applicable in drug screening and pharmacological analysis (Pinzi and Rastelli 2019). In our study, we utilized network pharmacology to predict potential targets of GE in improving atherosclerosis. Building upon the interactions among these potential targets, we further refined our selection to identify key targets. The majority of these key targets are closely associated with inflammation, oxidative stress, and apoptosis. Subsequent GO and KEGG enrichment analyses of these potential targets further validated these associations.

Based on the results of network pharmacology prediction, we selected some proteins closely related to oxidative stress, apoptosis, and inflammation as receptors for molecular docking with GE. The results revealed that GE exhibited excellent binding activity with these key target proteins, suggesting that these targets may play a pivotal role in the mechanism by which GE improves atherosclerosis. Furthermore, this underscores the need for experimental validation to confirm the reliability of the predicted results. Combining the outcomes of animal experiments with those of network pharmacology, we opted to use the H_2_O_2_‐induced HUVEC cell damage model for subsequent mechanistic exploration and validation.

Excessive or prolonged exposure to oxidative stress leads to endothelial dysfunction. H_2_O_2_ serves as the principal precursor for highly reactive oxygen species, including superoxide anions and hydroxyl radicals, that may induce oxidative stress‐induced damage and apoptosis within cells (Wilson and Keenan 2003). Our study showed that H_2_O_2_ induced cell apoptosis and ROS production in HUVEC cells and that GE significantly attenuated H_2_O_2_‐induced cell damage by decreasing the intracellular ROS overproduction, inhibiting the loss of mitochondrial membrane potential, and increasing the Bcl‐2/Bax ratio. When the Akt inhibitor (LY294002), GSK3β inhibitor (SB216763), and eNOS inhibitor (L‐NAME) were used to block the Akt/eNOS/NO and GSK3β/Nrf2/HO‐1 signaling, apoptosis and oxidative damage in HUVECs were increased. These results suggested that the protective effect of geranin on oxidative injury of endothelial cells is mediated by activation of the Akt/eNOS/NO and GSK3β/Nrf2/HO‐1 pathways.

As a highly diffusible signaling molecule, NO plays a role in maintaining cardiovascular function (Farah et al. 2018). But in the pathological condition of oxidative stress, excessive superoxide causes rapid oxidation and inactivation of NO, leading to endothelial dysfunction (Forstermann et al. 2017). Phosphorylation at Ser1177 of NO synthase (eNOS) plays an important role in nitric oxide (NO) production in endothelial cells (Zhang et al. 2019). Activation of kinases upstream of eNOS, such as Akt, can increase eNOS phosphorylation, improve endothelial function, and maintain blood–brain barrier function (Hong et al. 2019). In our study, GE pretreatment promoted NO release in H_2_O_2_‐induced HUVEC cells. The Western blot results showed that 8 μM GE significantly promoted the phosphorylation of Akt and eNOS, suggesting that GE can improve vascular endothelial injury through the Akt/eNOS/NO pathway. It is noteworthy that although both total Akt and p‐Akt expression increased significantly in GE‐treated HUVEC cells, the increase in p‐Akt protein expression was greater than that in total Akt. However, GE had no effect on the expression of total eNOS. These results indicated that GE may increase the level of p‐Akt by promoting both the expression and phosphorylation of Akt protein, and that the phosphorylated Akt directly acts on eNOS, increasing the phosphorylation level of eNOS.

Glycogen synthase kinase GSK‐3β, a highly conserved serine/threonine protein kinase, is implicated in the pathogenesis of diabetes, cancer, Alzheimer's disease, and atherosclerosis (Beurel et al. 2015; Lauretti et al. 2020). Given the critical role of GSK‐3β in regulating mitochondrial metabolism, we explored the mechanism through which GSK‐3β modulates the antioxidant response in vascular endothelial cells. Nrf2, a leucine zipper nuclear transcription factor, serves as a homologous substrate for GSK‐3β (Jiang et al. 2015). Phosphorylation of GSK‐3β induces Nrf2 phosphorylation, leading to Nrf2 nuclear export and proteasome degradation. In our study, GE pretreatment significantly increased the phosphorylation of GSK‐3β, which phosphorylated and promoted Nrf2 nuclear translocation, which then upregulated HO‐1 expression (Jain and Jaiswal 2007).

## 5 Conclusions

GE treatment significantly reduces serum lipids, oxidative stress damage, lipid deposition, and aortic plaque lesions associated with atherosclerosis in HED‐fed *ApoE* ^−/−^ mice. Network pharmacology analysis and in vitro experiments indicate that GE may alleviate atherosclerosis by inhibiting oxidative stress‐induced endothelial cell apoptosis and inflammation. Although geraniin, a polyphenolic compound derived from Geranium species, has been studied for various biological activities, its role in atherosclerosis has not been extensively explored. This study contributes new evidence of geraniin's potential to mitigate high‐fat diet‐induced atherosclerosis. While previous studies have explored the individual effects of various compounds (including polyphenols and other natural products) on atherosclerosis, this manuscript provides a more integrated and multi‐dimensional view of geraniin's effects. The combination of network pharmacology, molecular docking, and in vivo validation provides a stronger evidence base for geraniin's potential in atherosclerosis treatment compared to studies that rely solely on one of these methodologies.

## Author Contributions

**Yaoyao Xie:** conceptualization (equal), data curation (equal), formal analysis (equal), investigation (lead), methodology (equal), software (equal), visualization (lead), writing – original draft (equal). **Shihao Liu:** formal analysis (equal), investigation (equal), software (equal), visualization (equal). **Zhiheng Wei:** data curation (equal), formal analysis (equal), software (equal), validation (equal), visualization (equal), writing – original draft (equal). **Lisha Yu:** formal analysis (equal), investigation (equal), methodology (equal), validation (equal), visualization (equal). **Jianfeng Yu:** methodology (equal), resources (equal), validation (equal). **Lu Xu:** methodology (equal), validation (equal), visualization (equal). **Honglin Jiang:** conceptualization (supporting), writing – review and editing (equal). **Zhiliang Gu:** conceptualization (lead), funding acquisition (lead), methodology (equal), project administration (lead), resources (equal), supervision (lead), writing – original draft (equal), writing – review and editing (equal).

## Ethics Statement

This study was approved by the Institutional Review Board of Changshu Institute of Technology.

## Conflicts of Interest

The authors declare no conflicts of interest.

## Data Availability

All data presented in this study are available in the main body of the manuscript.
